# Collegiate Marching Band Artists Self-Report Previous Exercise-Associated Hyponatremia: A Cross-Sectional Study

**DOI:** 10.3390/nu17010079

**Published:** 2024-12-28

**Authors:** Dawn M. Emerson, Melani R. Kelly, Toni M. Torres-McGehee, Nancy A. Uriegas, Matthew O. Smith, Allison B. Smith, Kevin A. Kloesel

**Affiliations:** 1 Department of Exercise Science, Arnold School of Public Health, University of South Carolina, Columbia, SC 29208, USA; torresmc@mailbox.sc.edu; 2 Department of Exercise Science and Outdoor Recreation, College of Science, Utah Valley University, Orem, UT 84058, USA; melani.kelly@uvu.edu; 3 Department of Health and Human Performance, College of Education, Texas State University, San Marcos, TX 78666, USA; nau5@txstate.edu; 4 School of Music, University of Kansas, Lawrence, KS 66045, USA; maosmith@ku.edu; 5 Department of Health and Sport Sciences, Otterbein University, Westerville, OH 43081, USA; asmith1@otterbein.edu; 6 Drum Corps International, Inc., Indianapolis, IN 46241, USA; texasembassy@gmail.com

**Keywords:** performing arts, hydration, electrolytes

## Abstract

Exercise-associated hyponatremia (EAH) is commonly observed in endurance athletes, where prolonged physical exertion combined with being unaware of personal hydration needs can lead to excessive water consumption or inadequate sodium intake. Marching band (MB) is an emerging setting for sports medicine professionals. However, there is little research on non-musculoskeletal illnesses among these performing artists. This study is part of a larger cross-sectional study and seeks to examine whether MB artists have previously experienced EAH and the presence of known EAH risk factors (e.g., pre-existing medical conditions, medication use, nutritional behaviors). Active collegiate MB members (n = 1207; mean age = 19.6 ± 1.3 years) completed an online survey that characterized demographics, medical history, and nutrition behaviors. Statistical analyses included descriptives (mean, standard deviation) and frequencies for all data. Binomial logistic regressions analyzed the relationship and determined the odds ratio (OR) between previous EAH and EAH risk factors. Participants were from 23 different states, predominately White (82.9%), female (56.7%), and at NCAA Division I institutions (92%). A total of 74 (6.1%) participants reported previously experiencing EAH, 32 (43.2%) within the past year. Previous EAH occurrence was greatest among individuals who had kidney conditions (χ^2^(1) = 5.920, R^2^ = 0.013, OR = 15.708, *p* = 0.015); mood/neurological conditions (χ^2^(1) = 7.508, R^2^ = 0.017, OR = 2.154, *p* = 0.006), particularly anxiety (χ^2^(1) = 7.651, R^2^ = 0.017, OR = 3.590, *p* = 0.006); used mental health medications (χ^2^(1) = 7.512, R^2^ = 0.017, OR = 2.220, *p* = 0.006), particularly selective serotonin reuptake inhibitors (χ^2^(1) = 13.502, R^2^ = 0.030, OR = 3.297, *p* < 0.001); and who dieted (χ^2^(1) = 6.121, R^2^ = 0.014, OR = 1.913, *p* = 0.013) or perceived they had an eating disorder (χ^2^(1) = 9.502, R^2^ = 0.021, OR = 2.729, *p* = 0.002). Healthcare providers should be aware that MB artists may have pre-existing medication conditions, use medication, and have inadequate nutrition. Targeted hydration and nutritional education for MB artists is essential for effective prevention and early EAH recognition, improving health and performance for MB artists within demanding environments.

## 1. Introduction

Exercise-associated hyponatremia (EAH) is defined by a blood sodium concentration [Na^+^] < 135 mmol/L that develops either during physical activity or within 24 h after activity. EAH signs and symptoms range from mild (e.g., headache, dizziness, nausea) to severe (e.g., altered mental status, vomiting, seizure). Severity depends on how much and how rapidly [Na^+^] decreases from the individual’s baseline values [1]. When individuals lack clinically relevant signs or symptoms, it is termed biochemical or asymptomatic EAH [2,3,4]. Therefore, EAH diagnosis is based on both low blood [Na^+^] and exhibiting signs and symptoms. Misdiagnosing or mistreating EAH can have potentially life-threatening consequences due to associated cerebral and/or pulmonary edema [1].

EAH has traditionally been observed among athletes engaged in activity lasting longer than 4 h, such as ultra-marathon and marathon runners [5,6], triathletes [7,8], and ultra-cyclists [3,4]. However, what was once a concern primarily among endurance athletes has now extended across a broader spectrum of physical activities. EAH is observed in high school football [9], shorter distance running [2], cycling [10], hiking [11], yoga [12], and military training [13,14]. Frequently, EAH is caused by a combination of prolonged activity and either modifiable or non-modifiable factors. Non-modifiable factors are outside the person’s control (e.g., chronic medical condition, prescription medication use), while modifiable factors are within the person’s control (e.g., using supplements, hydration status).

A primary modifiable factor for EAH is fluid consumption, where individuals drink hypotonic fluids in excess of fluid lost via sweat, respiration, and urine production [1]. Misconceptions about the importance of hydrating to limit health and performance detriments lead to individuals overconsuming fluids before, during, or after activity. Further, inexperience or inadequate training may be another factor [1]. Not only are individuals not educated about their individual hydration needs, they may not be appropriately conditioned for the activity they are trying to perform.

There is conflicting research on some EAH risk factors, specifically sex, medication use, and pre-existing medical conditions. Females have displayed greater EAH risk attributed to smaller body mass and slower pace compared to males [5,7]. However, Almond et al. [6], who corrected for body mass index and race time, found no difference in EAH incidence between sexes. Over-the-counter (OTC) medications, like non-steroidal anti-inflammatory drugs (NSAIDs), promote water retention hours after exercise cessation by impairing free water clearance [15], supporting investigators who have found an association between NSAIDs and EAH [5,8]. Conversely, other investigators report that NSAIDs do not increase EAH risk [6,16]. Determining how pre-existing medical conditions affect EAH incidence is challenging due to the lack of medical history documentation in endurance races.

To understand how medical conditions and medications may promote EAH, it is essential to understand the different mechanisms for developing EAH. While an individual may have normal plasma volume during exercise, exercise-induced factors like nausea and vomiting [17], hyperthermia [18], inflammatory cytokine release [19], and/or hypoglycemia [20] can stimulate anti-diuretic hormone (ADH) release, reducing renal water excretion [21]. Mental health medications, specifically selective serotonin reuptake inhibitors (SSRIs), are associated with the syndrome of inappropriate ADH secretion (SIADH) [22]. SIADH promotes EAH through continuous ADH release, resulting in water retention and subsequent decreases in blood [Na^+^] [23]. Individuals with renal or cardiovascular diseases may have a predisposition to EAH due to pathophysiological alterations affecting fluid/electrolyte balance regulation or associated medications (e.g., hypertension drugs) [24] that alter fluid regulation. EAH risk increases if these individuals with pre-existing medical condition(s) or who use medication(s) that affect ADH also consume excessive hypotonic fluids.

Marching band (MB) is a popular high school and college activity, with bands ranging from 10 to more than 300 members. MBs typically include 4 sections: (1) woodwind (clarinet, piccolo, saxophone, etc.), (2) brass (trumpet, trombone, mellophone, sousaphone, etc.), (3) drumline (bass, quads, snare, etc.) and pit (marimba, xylophone, chimes, etc.), and (4) auxiliary (color guard, dancers, twirlers, drum majors). MB contributes to daily physical activity levels. Depending on activity type, metabolic demands can vary 3 to 8 metabolic equivalents, and heart rates can exceed 160 beats per minute [25,26]. MB artists perform at football games, competitions, and parades. MB artists march at slow to fast tempos (e.g., 132 steps per minute) and must play, dance, or twirl, sometimes for 2–6 h or longer [26].

There is no governing body for college MBs; therefore, rehearsal duration, frequency, and intensity are dependent on the institution. Typically, MB members start with band camp in late July/early August, when temperatures are usually hot and humid. Band camp lasts about 7–14 days and often includes multiple daily outdoor rehearsals lasting over 2 h. School-year rehearsals may range from 1–3 h, either daily or 3–4 days a week. In addition to environmental stress and physical exertion, MB artists wear uniforms that often cover their entire body. Added clothing creates a barrier to sweat evaporation, a key mechanism for losing metabolic heat during activity, subsequently increasing core body temperature [27] and the risk for exertional heat illness (EHI).

Research on medical conditions among MB artists is limited. However, it is known they sustain musculoskeletal injuries (e.g., sprains, stress fractures) [28,29,30] and non-musculoskeletal illnesses, such as EHI, asthma attacks, skin reactions, etc. [28,29,30]. Unfortunately, the prevalence of specific non-musculoskeletal conditions while participating in MB remains unclear, as these conditions are frequently grouped under “general medical conditions”. MB artists are often considered part of the general student population, typically lacking pre-participation examinations to identify underlying health conditions that may predispose them to adverse medical events during physical activity. The limited physiological studies on MB artists show individuals experience hyperthermia (core body temperatures > 40 °C) [25] and hypohydration [25,31] during activity. Further, MB artists have been identified to be participating with ongoing medical conditions, including mental health and current illness (e.g., cold) [25].

Most MBs do not have access to on-site healthcare providers, such as athletic trainers, who are trained in the prevention, recognition, and management of musculoskeletal and non-musculoskeletal conditions. MB is an emerging setting for healthcare providers; therefore, there is a need for specialized, evidence-based care and further research to better understand the medical conditions MB artists experience. The purpose of this study is to explore whether collegiate MB artists have previously experienced EAH during activity and what risk factors may be associated with these occurrences. Regarding risk factors, it was hypothesized that using mental health medications, engaging in pathogenic nutrition behaviors, and having underlying renal conditions would be associated with EAH.

## 2. Materials and Methods

### 2.1. Participants

A total of 1207 MB artists from National Collegiate Athletic Association (NCAA) Division I, II, and III; National Association of Intercollegiate Athletics (NAIA); and National Junior College Athletic Association (NJCAA) institutions participated. To be included, MB artists were active members of their institutions’ MB during the fall 2019 football season and were between 18 and 35 years old. The study was approved by the University of Kansas (STUDY00142728) and the University of South Carolina (PRO00081581) Institutional Review Boards.

### 2.2. Instrumentation

The present study is part of a larger study, which seeks to examine pre-existing medical conditions, previous self-reported EHI occurrence, and risk factors for EHI among collegiate MB artists [32]. Specific to this study, questions on EAH were included. A description of EAH, including etiology and signs and symptoms, was provided to participants, followed by a question asking whether the individual had previously experienced EAH. If “yes”, the individual was asked to report the date of the most recent occurrence.

The electronic survey was developed in Qualtrics (Provo, UT), consisting of 75 multiple-choice, multiple-selection, and manual-entry questions. Questions assessed demographics, medical history, medication and supplement use, and nutritional behaviors. Demographics and descriptives included age, race, sex, college classification (e.g., freshmen), years of MB experience, instrument/auxiliary section, collegiate division (e.g., NAIA), and state their institution was located in. Using state and conference information, participants were categorized into heat safety regions 1 (cooler), 2, or 3 (hotter) based on Grundstein et al. [33].

Medical history questions were based on the Physical Activity Readiness Questionnaire for Everyone, a self-administered pre-participation risk screening tool to assess physical activity and exercise participation [34]. For medication and supplement use, participants were asked the type and dose of prescribed and OTC medications and types of supplements currently used. For nutritional behaviors, questions were based on recommended screening items to include during pre-participation examinations [35]. Participants were asked if they limited their food intake. If “yes”, participants were asked the methods (e.g., skip meals). Participants reported eating disorder (ED) risk behaviors (e.g., laxative use, vomiting) and whether they have been diagnosed with or think they have an ED. Participants were asked if they gain or lose weight for performance, whether anyone recommended they change their weight and eating habits, if anyone has set a target weight for them, and about exercise habits outside scheduled MB activities. An additional description of the survey has been previously published [32].

### 2.3. Study Procedures

Participants were recruited in 2 phases. First, a convenient sample of collegiate MB artists was contacted through an email sent to 3 NCAA Division I universities’ MB directors, who distributed the anonymous, shareable survey link to their MB artists. Second, to increase participation, an email was sent to MB directors across the U.S. through the College Band Directors National Association. Directors were informed of the study’s purpose and provided the survey link to distribute to their MB members. Interested participants clicked the survey link for additional study information. Individuals provided written informed consent by selecting “yes” and then proceeded to the survey. If an individual did not consent, they selected “no” and did not proceed. No identifying questions were asked; therefore, no follow-up emails were sent. The survey was open from August to December 2019.

### 2.4. Statistical Analysis

IBM SPSS Statistics (version 27; IBM Inc., Armonk, NY, USA) was used for all analyses. The significance level was set at *p* < 0.05. A-priori power with a 95% confidence level and 3% error indicated that 1014 responses were necessary to achieve appropriate statistical power. Descriptive statistics [mean (standard deviation)] were calculated for all data. Cross-tabulations were used to determine frequency across categories. Binomial logistic regressions analyzed the relationship and determined the odds ratio (OR) between previous EAH and EAH risk factors. Based on existing EAH literature [1,6,8,36], the primary investigators identified risk factors and categorized them into either non-modifiable: age, sex, race, MB section, years of experience, college classification, collegiate division, state, pre-existing medical conditions, prescription medication, pressures to change weight, and target weight set/routine weigh-ins, or modifiable: OTC medications, supplements, exercise outside MB, following a diet, and weight loss methods (diet pills, laxatives, exercise, diuretics, vomiting). Standardized residuals, Cook’s D, and leverage points were examined for potential outliers and influential values that may have affected the regression results; no data were removed. Nagelkerke R^2^ values are reported.

## 3. Results

### 3.1. Demographics

A total of 1572 MB artists started the survey; 1213 completed the survey. No follow-up recruitment was necessary due to achieving the desired response rate. Six participants reported being under 18 years old and were removed from the final sample. Our final sample size was 1207 MB artists (76.8% completion rate). Demographics for the overall sample are previously published [32]. Overall, participants’ mean age = 19.6 ± 1.3 years, were predominately White (82.9%), from NCAA Division I institutions (92%), and had more than 6 years of experience marching (59.1%). Participants were from 23 states and predominately in heat safety region 3 (45.8%). Auxiliary (n = 125) was predominately color guard (76.8%, n = 96). For the present study, Table 1 shows the frequency for sex, race, instrument section, MB experience, college classification, and physical activity outside MB for those reporting previous EAH.

### 3.2. EAH Occurrence

**Figure 1:**
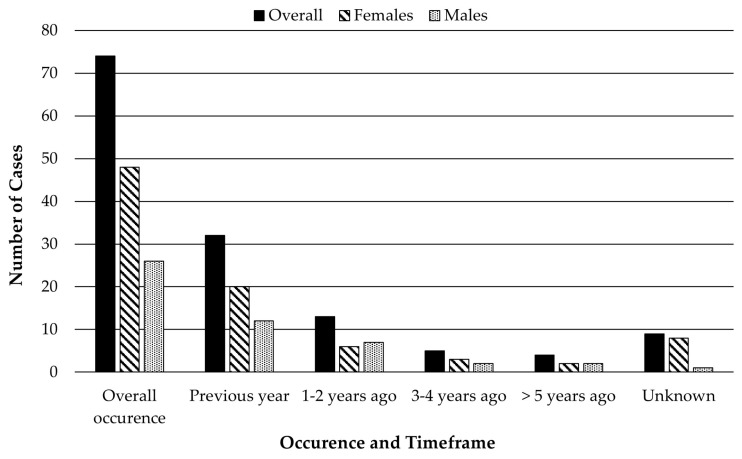
Exertional hyponatremia occurrence overall and by sex.

Figure 1 shows the frequency of previous EAH reported and when the EAH occurred (e.g., previous year) overall and across sex. A total of 74 (6.1%) MB artists reported previously experiencing EAH; 32 (43.2%) occurred in the previous year. Using college classification and when the EAH occurred, 34/74 individuals (46%) experienced EAH in college, while 13 (17.6%) were in high school. Whether EAH in the previous year was during college or high school was unable to be determined for 9 college freshmen (12.2%). More females reported previous EAH than males (Figure 1, Table 1); however, there was no statistically significant association.

### 3.3. Risk Factors

#### 3.3.1. Modifiable Risk Factors

Nearly 42% of those reporting previous EAH participated in some physical activity outside MB, predominately endurance training (Table 1). However, regularly exercising outside of MB activities was not associated with previous EAH. Nutritional behaviors, OTC medications, and supplement use frequency and associations to EAH are presented in Table 2. Previous EAH was significantly associated with perceived ED, dieting, and using diet pills to change weight. Taking any OTC medication approached significance (*p* = 0.060), and no subcategories of OTC medications, including NSAIDs, were significantly associated. Due to the low n for individual supplements, several supplements were combined into larger categories. For instance, using protein powder, creatine, caffeine, and/or amino acids were categorized into “performance” supplements. Performance supplements and vitamins/minerals were not significantly associated with previous EAH, while “other” supplements (e.g., digestive support, omega-3) were (Table 2).

#### 3.3.2. Non-Modifiable Risk Factors

Frequency and associations to previous EAH for mental health conditions and using mental health drugs are presented in Table 3. Taking any mood or neurological drug and mood only were significantly associated. For specific drug types, anti-anxiety and anti-depressants were associated with EAH, which aligns with the higher odds among individuals with anxiety and depression (Table 3). The highest odds occurred for SSRIs. Taking any mood or neurological medication was also significantly associated with experiencing EAH within the previous year (χ^2^(1) = 5.459, R^2^ = 0.021, OR = 2.65, 95% CI = 1.23–5.70, *p* = 0.019). Of the 32 EAH cases in the previous year, 10 (31.3%) were among people taking any mental health drug. All 10 individuals were taking SSRIs (χ^2^(1) = 12.740, R^2^ = 0.048, OR = 4.83, 95% CI = 2.23–10.49, *p* < 0.001).

Table 4 presents frequency and associations for other non-modifiable risk factors, including pre-existing medical conditions, prescription medication use, and nutritional behaviors. Previous EAH was significantly associated with having any cardiovascular condition, using cardiovascular medication, and previous EHI (exertional heat exhaustion and/or exertional heat stroke). Those with kidney conditions were 15 times more likely to have previously experienced EAH. No other prescription medications or nutritional behaviors were significant.

Regarding descriptive variables (Table 1), sex, race, MB experience, and college classification were not significantly associated with previous EAH. Similar to the overall study population, individuals previously reporting EAH were predominately at NCAA Division 1 institutions (89.2%, n = 66) and in region 3 (54.1%, n = 40); these factors were not significantly associated with EAH occurrence. Previous EAH was significantly associated with MB section (χ^2^(1) = 7.811, R^2^ = 0.017, OR = 1.057, 95% CI = 1.02–1.10, *p* = 0.005). Although there were more overall occurrences reported among woodwind and brass compared to auxiliary and drumline/pit (Table 1), auxiliary exhibited greater odds for EAH than musicians (χ^2^ = 8.652, R^2^ = 0.019, OR = 2.59, 95% CI = 1.44–4.66, *p* = 0.003). Further examination of instrument type (e.g., piccolo, clarinet, trumpet) did not reveal any significant associations for EAH occurrence. Approximately 90% of the auxiliary were females (n = 113), and of the 16 auxiliary reporting previous EAH, 12 (75%) were female. Within the woodwind section, 20/28 EAH occurrences (71.4%) were females.

Given the greater odds and higher overall occurrence for females, significant EAH risk factors were further analyzed to determine potential sex differences. Frequency and regression analyses for sex within the entire study population are presented in Table 5. Females exhibited higher frequency and odds for mental health conditions, using mental health medications, previous EHI, perceiving they have an ED, and using dieting or diet pills to control their weight.

## 4. Discussion

This study is the first to investigate collegiate MB artists’ pre-existing medical conditions, specifically whether artists have previously experienced EAH and associated risk factors. Seventy-four participants had previously experienced EAH, either in high school or college. Our study adds to the existing literature [9,10,13] that EAH affects physically active individuals outside the traditional endurance athlete. Typically, EAH is associated with overzealous drinkers, females, “salty sweaters”, prolonged activity, weight gain during activity, lack of physical fitness, and lack of heat acclimatization [1,5,6,36]. Due to the retrospective nature of our study, we were unable to examine how factors such as heat acclimatization status, physical fitness level, weight gain, or fluid consumption influenced participant’s EAH occurrence. The study was able to identify that previous EAH was associated with mental health, cardiovascular conditions, and nutrition behaviors.

### 4.1. Modifiable Risk Factors

While fluid overconsumption was beyond the scope of our study, previous studies show that MB artists tend to consume less fluids during rehearsals and games than their sweat rates require [25]. MB artists often arrive to rehearsals euhydrated [25,31] but are more often hypohydrated on game days and drink more fluids on these long days [25]. Hypohydration exceeding >2% of body mass leads to increased cardiovascular strain [37] and can cause EHI symptoms (e.g., headache, fatigue, nausea). Unfortunately, without proper education or knowledge of their personal fluid needs, individuals may try to mitigate their dehydration/EHI risk by overconsuming fluids or “hyperhydrating”. This was the case in several non-endurance EAH cases, including in football [9], yoga and interval training [12], military training [13,14], and other moderate exercise bouts [11]. Excessive fluid consumption is a key EAH factor [1]; therefore, prevention strategies must focus on educating individuals about proper hydration.

This study did not find using OTC medications was associated with previous EAH. There are potentially two explanations for this. First, the survey question asked participants about their current use of OTC medications, which may have differed from when their EAH occurred. OTC medications are often used short-term and as needed compared to prescription medications, which are more likely to be used long-term to manage a medical condition. Second, the number of individuals reporting NSAID use was low (n = 7 for OTC and prescription combined). While the role of NSAIDs as a risk factor for EAH has been debated, evidence exists that they may contribute to EAH [5,8]. Similarly, the number of participants reporting supplement use was low; however, taking “other” supplements was associated with previous EAH. Eight individuals were in the “other” category and took a variety of supplements, including digestive support, fish oil/omega-3, melatonin, powdered greens, and apple cider vinegar pills. Despite the lack of association with previous EAH, the study provides evidence that MB artists are taking OTC medications and supplements while performing MB activities. This is cause for concern, as a wide variety of supplements are available on the market with limited regulation over their quality and content. Active individuals such as MB artists should carefully consider the potential risks and efficacy of nutritional supplements prior to use.

Nutritional behaviors are complicated, being both modifiable and non-modifiable factors depending on cause and co-morbidities. For example, individuals may choose to purge in an attempt to control their weight, which may be due to internal pressures such as body image dissatisfaction or external pressures from coaches, parents, or peers to be a certain weight [38,39]. This study is consistent with previous research that identified that MB artists engage in pathogenic behaviors [39,40]. These behaviors can result in inadequate nutrition intake or a state of low energy availability. Low energy availability occurs when individuals do not have adequate energy intake to match the demands of daily life after activity and is a primary component of the male and female athlete triads and relative energy deficiency in sport [41]. There are potentially serious health consequences to the triad or relative energy deficiency, including cardiovascular complications (e.g., hypotension, arrhythmias, bradycardia, cardiac muscle wasting) and renal dysfunction (acute kidney injury, decreased glomerular filtration, decreased renal blood flow, metabolic acidosis/alkalosis) [41,42,43]. Behaviors such as vomiting and diuretic use can also cause water and electrolyte imbalances (e.g., hypovolemia, hypokalemia, hypocalcemia) [42]. Overall, pathogenic behaviors can lead to vitamin and mineral deficiencies, including low sodium, that perpetuate EAH, particularly if combined with high water intake during activity.

### 4.2. Non-Modifiable Risk Factors

Most field studies on EAH do not include information on the participants’ health status prior to the incident, such as pre-existing medical conditions or medication use beyond NSAIDs [3,5,6,7,8,44,45]. However, some insight can be gained through military data. O’Brien et al. reported 7/12 soldiers to be in “good general health” prior to their EAH, while one soldier was actually excluded from data analysis because they had an acute respiratory illness prior to their incident [14]. Another EAH case during military training occurred concurrently with exertional heat stroke and was in an individual with no pertinent medical history and not taking medications and supplements [13]. Similarly, 5/7 patients engaged in different modes of moderate exercise had no pertinent medical history, while 1 reported seasonal allergies and another a renal condition [11].

To better understand how pre-existing medical conditions and medications can contribute to EAH, it is important to look at the etiology of other types of hyponatremia. Hypovolemic hyponatremia occurs due to a loss of fluid and sodium, perhaps caused by diuretic use, mineralocorticoid deficiencies, gastrointestinal pathology, or renal dysfunction. Hypervolemic hyponatremia is associated with congestive heart failure and renal disease, where the kidneys are unable to excrete water and fluid builds up. Euvolemic hyponatremia is the most common type and is observed in patients with chronic medical conditions or who have been hospitalized. Causes of euvolemic hyponatremia include inappropriate and persistent ADH stimulation (i.e., SIADH), disease, and medications [23].

ADH is produced by hypothalamic neurons, extending into the posterior pituitary, where the hormone is stored until needed. ADH release is primarily triggered by increased plasma osmolality, specifically [Na^+^], and decreased plasma volume or arterial pressure. When plasma osmolality indicates a hydrated state and a person consumes fluid, excess water is excreted. When plasma osmolality levels indicate dehydration or baroreceptors sense lowering blood pressure, ADH causes the kidneys to retain fluid and help maintain physiological function. Concurrently, thirst is stimulated, and the person consumes fluids to help increase blood pressure or lower osmolality [46]. In the case of someone experiencing SIADH, the perpetual release of ADH causes fluid retention regardless of plasma volume/osmolality levels [23]. ADH and SIADH can also be triggered by pain, nausea, and other physical and emotional stressors via the limbic system and hypothalamus [21].

Regarding mental health, performing artists are often at higher risk for depression and anxiety than the general population [47,48]. Within our larger study, 208 MB artists (17.2%) reported having a mental health condition [32]. More than 78% of gender-diverse MB artists (n = 78) exhibited anxiety and/or depression risk [48]. Specific to this study, those with anxiety and taking anti-anxiety medications were 3–3.5 times more likely to report previous EAH. Similarly, MB artists with depression were 2.7 times more likely, and those taking SSRIs exhibited the highest odds, being 3.3 times more likely to report previous EAH. Our data support previous case studies associating mental health drugs with hyponatremia [22,49], though the cases were in elderly individuals and euvolemic hyponatremia (not exercise-associated). There are several theories regarding how mental health drugs stimulate ADH production and excretion; a particularly relevant theory is that the medications affect serotonin receptors, which stimulate ADH [50]. Other potential mechanisms include pathophysiological and medication influences on the gut/brain axis, renin/angiotensin aldosterone system, hypothalamus, or limbic system [51,52].

The association between previous EAH and cardiovascular and kidney disease is not surprising, given the effects these conditions have on blood flow, kidney function, and fluid/electrolyte balance. Cardiovascular medication was not significantly associated, but these medications (e.g., angiotensin-converting enzyme inhibitors, sodium or potassium channel blockers) are known risk factors for hyponatremia due to either direct effects on kidney function or hormonal regulation [23,24]. Thus, although the mechanism between specific medications and EAH risk is not elucidated, it is plausible that individuals using drugs that alter ADH secretion, or fluid/electrolyte balance in general, are at greater EAH risk.

Among the larger study population, 35 participants reported being diagnosed with an ED [32], and 5 of those individuals reported a previous EAH. Additionally, 16 of the 120 participants who perceived they had an ED [32] also reported a prior EAH. Relying solely on ED diagnosis as an identifiable risk factor may cause undiagnosed individuals to be overlooked. Patients with EDs may experience hyponatremia due to overconsuming water instead of food, in part to curb hunger feelings [42,53]. Other ED patients may develop hyponatremia when overconsuming water to give the perception of gaining weight or meeting a goal weight when they visit a healthcare provider [42,53]. Collegiate MB auxiliary and musicians are at risk for EDs and engage in pathogenic behaviors to control weight, with females engaging in purging and laxative use more significantly [40]. Given the consequences of malnutrition on electrolyte balance and renal function, MB artists with perceived or diagnosed ED should have access to medical professionals who can discuss nutrition and overall wellness to minimize the risk for EAH and other medical issues.

Although not statistically significant, almost twice as many females reported previous EAH than males. MB artists, regardless of sex, have similar physical demands during rehearsals and performances, are exposed to similar environmental conditions, and are participating for similar durations. Therefore, potential differences between sexes are more likely related to other factors such as pre-existing medical conditions and medications. Greater odds for EAH occurrence among the auxiliary could be due to females significantly reporting more mental health conditions, taking more mental health medications, engaging in dieting, and perceiving they have an ED. As previously mentioned, MB artists across all sections and sexes exhibit risk for ED [40], depression, and anxiety [48]. Our results did not find that pressure to change weight or meet a certain weight was associated with previous EAH, yet prior research has explored some unique risk factors that could explain the higher odds among the auxiliary. Band personnel, sports spectators, and society can place emphasis on auxiliary members’ physical appearance and body size, particularly when wearing potentially revealing or tight uniforms. These pressures can cause the individual to become preoccupied with their own appearance and weight, leading to or amplifying pre-existing ED or pathogenic behavior [39,54].

Finally, high environmental temperatures can contribute to EAH, but EAH occurs across a wide range of temperatures, from cool to hot conditions (52–64 °F to 91.5–93.9 °F) [2,3,8,14]. MB artists were asked to report where their school was located, and a majority were located in the hotter region 3 of the U.S. Our study was unable to draw any conclusions from this because we did not specifically ask what state or region the individual had experienced their EAH. College MB artists are exposed to extremely hot environmental conditions, reaching 110.6 °F and 123.8 °F wet bulb globe temperature during rehearsals and games, respectively [25]. A case series examining news reports of MB EHIs found temperatures ranging from 73 °F to 111 °F [55]. High environmental temperatures increase sweat rate [56] and can lead to ADH secretion as the cardiovascular system attempts to maintain plasma volume and blood pressure during activity [18,36]. Being unacclimatized to the heat is a potential risk factor because of higher sweat sodium losses. Heat acclimatization increases sweat losses yet decreases sweat sodium concentration to help preserve sodium and fluid. To limit EAH and EHI risk, individuals should gradually acclimatize to the environment and physical demands while being aware of their personal fluid needs [56].

### 4.3. Clinical Implications

Despite the physical strain associated with MB participation, MB directors often face unique challenges in identifying and managing risk factors associated with EAH compared to college athletic settings. The NCAA, NAIA, and NJCAA require student-athletes to complete pre-season screening to ensure safe participation in the desired activity. Without a governing body, any policies to minimize health risks for college MB artists are at the discretion of individual institutions. Some institutions require MB artists to complete a pre-participation examination, which is administered by a medical professional who can screen for risk factors and ensure appropriate care (e.g., testing, medication management, accommodations) prior to participation. At other institutions, artists are only required to self-report health information through a screening form before they can participate, and many institutions do not collect any health information. Self-reported forms are not necessarily screened by medical professionals, and band personnel are likely not educated on how to identify, educate, and manage an artist who may be at higher risk for experiencing an adverse medical event (e.g., asthma attack, EHI, EAH, hypoglycemia, seizure) during MB activities. Requiring MB artists to complete pre-participation physical exams or screenings prior to pre-season rehearsals (band camp) is a critical component in mitigating risks during MB.

While medical coverage is required for college athletic participation, access to coverage for MB activities varies significantly. Some institutions provide athletic training coverage for MBs through athletics or student recreation departments; however, many MBs do not have access to these resources. In addition to medical care, athletic trainers can provide and coordinate ongoing medical care, provide education to MB directors and artists, and create medical policies and procedures, including emergency action plans. Educational sessions can be held for MB personnel in leadership roles (i.e., band directors, graduate assistants, section leaders, drum majors) on proper hydration and nutrition strategies, recognizing signs and symptoms of EAH, and procedures to manage an individual who is suspected to be experiencing EAH. Educating the leadership can help improve the overall health and safety of the entire band. If a MB does not have consistent access to an athletic trainer or other healthcare provider, MB directors may be able to develop a relationship with an athletic trainer or other qualified healthcare provider within their institution or community for guidance on medical policies and procedures.

For healthcare professionals working with MBs, education pertaining to individual hydration requirements and strategies should be a key area of emphasis. Educating artists on appropriate fluid replacement may prevent inadequate or excessive fluid consumption before, during, or after activity, which can lead to hypohydration or contribute to EAH, respectively. Determining a person’s sweat rate can help establish how much fluid the individual should consume during MB activities, with consideration to environmental strain and activity intensity. Urine color, body weight, and thirst can be easy self-assessment tools for MB artists to determine their hydration status daily. Drinking to thirst combined with maintaining a urine color around 4 will help prevent overhydration [56,57]. A key factor associated with EAH is gaining weight during activity, which suggests the individual is overcoming fluids [36]. Maintaining pre- to post-activity body mass loss of around 1–2% will prevent health detriments associated with hypohydration and reduce the risk of overhydrating [56]. Daily weighing is not advised for individuals with diagnosed ED, body dissatisfaction, or a preoccupation with weight, as a focus on body composition can increase risk or complications associated with their condition [54]; therefore, alternative hydration measures (urine and thirst) should be used among these individuals.

An emphasis should also be placed on preventing EAH through nutrition education. Understanding beverage composition and electrolyte supplements can help individuals choose appropriate strategies. Most sports beverages (e.g., Gatorade, Powerade, Pedialyte) contain electrolytes but are hypotonic compared to blood and are not found to decrease EAH risk [36]. When activity is 1 h or less, water is typically sufficient to maintain hydration. For longer-duration activities, individuals may benefit from sports beverages, in large part to replenish carbohydrates [56]. The added salt in a sports beverage may stimulate the thirst mechanism, and flavoring can encourage the participant to drink more fluids to maintain hydration. Recommended daily sodium intake is 2300 mg, which is generally not a concern for healthy active individuals eating a well-balanced diet. Individuals who are “salty sweaters” (large sodium losses in sweat), diagnosed with EDs, engage in disordered eating behaviors (e.g., vomiting), or who use diet to either control weight or maintain health (e.g., reduce hypertension) may be cutting sodium voluntarily or involuntarily.

### 4.4. Limitations and Future Research

The data in this study were self-reported, and researchers assume participants correctly interpreted questions. It is possible that participants may have misinterpreted the definition of EAH and reported EAH instead of EHI. However, it is worth noting that when conducting robustness tests on significant associations, the association between EAH and anti-depressant medication, SSRIs, and perceiving an ED all remained whether the person reported having a previous EHI or not. To enhance the accuracy of EAH prevalence rates, future researchers should work with an athletic trainer or physician providing care to MBs and, together, track exposure and diagnosed EAH occurrences throughout the season.

The small number reporting previous EAH and even smaller numbers in some risk factor categories (e.g., supplement use, NSAIDs) may have prevented us from finding associations. Additionally, where significant associations occurred, there may be some limits to the reliability; this is likely the situation for kidney disease. More research is needed to examine EAH risk factors, particularly pre-existing medical conditions, medications, supplements, and nutrition behaviors. These risk factors could be identified on pre-participation screening, medical records, or an additional survey with more detailed questions about the individual’s medical history. For patients diagnosed by a healthcare provider with EAH, a follow-up with the MB artist, either through surveys or an interview, would allow researchers to specifically identify the events and factors that may have perpetuated the EAH.

Most participants were from NCAA Division I, Power 5 (at the time of the study) institutions. The initial study recruitment was specifically to 3 NCAA Division 1 schools, yet this yielded only 180 responses. The subsequent survey distribution went to a broader population. Band directors then sent the survey link to their own MB members. Our high response rate from MB artists, predominately in hotter regions and at large schools, may have been due to band directors and artists having a personal interest in the survey topic, where they may have self-selected to complete the survey if they had previously experienced issues with exercising in the heat. To improve the understanding of EAH in MB, future research should include high school, elite (Drum Corps International^®^), military, and smaller college MBs.

Due to an unintentional error, participants during the second round of recruitment were not asked to self-report height and weight, which prevented determining if body size was a risk factor. It is imperative future research evaluate physiological measurements (e.g., plasma [Na^+^], plasma volume, body composition), environmental stress, and activity (e.g., intensity, duration) to better understand the physical demands and potential mechanisms for EAH among MB artists.

## 5. Conclusions

The first purpose of this study was to explore whether collegiate MB artists had previously experienced EAH. This objective was met, identifying that 6.1% of the sample population had experienced EAH. Secondly, the study examined which risk factors could be associated with EAH. The hypothesis was partially confirmed. Using mental health medications, specifically SSRIs and anxiolytics, and engaging in pathogenic nutrition behaviors was significantly associated with previous EAH. Pre-existing cardiovascular and renal disease was also significant. While sex was not a statistically significant factor, the study identified a higher frequency overall for females and within the auxiliary (predominately female), which may be attributed to the higher reported mental health conditions and use of pathogenic nutritional behaviors among females.

Currently, the presence of policies and medical coverage to improve college MB artists’ health and safety is at the discretion of individual institutions. MB directors should work with athletic trainers to establish specific policies and procedures to prevent, identify, and manage EAH. Together, directors and healthcare providers can reduce EAH risk by implementing mandatory pre-season screening (before band camp) by a healthcare provider and incorporating hydration and nutrition education sessions for MB artists.

## Figures and Tables

**Table 1:** Descriptives of MB artists who self-reported previous exercise-associated hyponatremia (EAH).

	N (%) (x/74 EAH)
**Sex ^a^**	
Male	26 (35.1)
Female	48 (41.9)
**Race**	
Asian	2 (2.7)
Black or African American	3 (4.1)
Hispanic or Latino	2 (2.7)
White	58 (78.4)
Multi-racial	6 (8.1)
Other ^b^	3 (4.1)
**Instrument Section ^c^**	
Woodwind	28 (37.8)
Brass	26 (35.1)
Drum/Pit	4 (5.4)
Auxiliary	16 (21.6)
**MB Experience (years)**	
<1	1 (1.4)
2–3	2 (2.7)
4–5	22 (29.7)
6–7	34 (45.9)
>8	15 (20.3)
**College Classification**	
Freshmen	25 (33.8)
Sophomore	16 (21.6)
Junior	12 (16.2)
Senior	21 (28.4)
**Physical Activity Outside MB**	31 (41.9)
Endurance training	19 (25.7)
Resistance training	13 (17.6)
Sport/Recreational activity	6 (8.1)
Workout class	3 (4.1)
Other	4 (5.4)

Abbreviations: MB = marching band. ^a^ No reported EAH among individuals reporting sex as male-to-female, female-to-male, or prefer not to disclose. ^b^ Other includes American Indian or Alaska Native, Native Hawaiian or Pacific Islander, prefer not to say, and Jewish. ^c^ Significant association across MB section.

**Table 2:** Frequency of modifiable risk factors and associations to previously experiencing EAH.

	N (%) (x/74 EAH)	χ^2^	R^2^	OR	95% CI	*p* Value
**Nutritional behaviors**						
Perceived ED	16 (21.6)	9.502	0.021	2.729	1.51–4.92	**0.002**
Dieting	26 (35.1)	6.121	0.014	1.913	1.16–3.15	**0.013**
Behaviors to change weight						
*Diet Pills*	7 (9.5)	9.802	0.022	1.498	1.20–1.87	**0.002**
*Diuretics*	1 (1.4)	0.606	0.001	1.370	0.67–2.79	0.436
*Exercise*	29 (39.2)	1.234	0.003	1.057	0.96–1.16	0.267
*Laxatives*	1 (1.4)	0.024	0.000	1.086	0.39–3.02	0.877
*Vomit*	5 (6.8)	2.027	0.005	2.146	0.82–5.63	0.155
**Any OTC medication**	23 (31.1)	3.542	0.008	1.660	1.00–2.77	0.060
Acetaminophen	2 (2.7)	1.643	0.004	3.119	0.67–14.50	0.200
Allergy	14 (18.9)	1.570	0.004	1.495	0.82–2.74	0.210
Gastrointestinal	3 (2.0)	1.358	0.003	2.237	0.65–7.68	0.244
NSAIDs	5 (6.8)	0.238	0.001	1.273	0.50–3.27	0.626
**Any supplement**	26 (35.1)	1.596	0.004	1.382	0.84–2.27	0.206
Performance ^a^	3 (4.1)	0.455	0.001	1.554	0.46–5.21	0.500
Vitamins or minerals	19 (25.7)	0.168	0.000	1.120	0.65–1.92	0.682
Other ^b^	8 (10.8)	4.587	0.010	2.572	1.17–5.64	**0.032**

Abbreviations: CI = confidence interval; EAH = exercise-associated hyponatremia; ED = eating disorder; NSAID = non-steroidal anti-inflammatory drug; OR = odds ratio; OTC = over-the-counter. Note: Bold denotes significant *p* values. ^a^ Includes protein powder, creatine, caffeine, and amino acids. ^b^ Includes digestive support, fish oil/omega-3, melatonin, powdered greens, and apple cider vinegar pills.

**Table 3:** Frequency and associations for mental health conditions and medications for individuals previously experiencing EAH.

	N (%) (x/74 EAH)	χ^2^	R^2^	OR	95% CI	*p* Value
**Any Mental Health Condition**	22 (29.7)	7.508	0.017	2.15	1.28–3.63	**0.006**
Anxiety	8 (10.8)	7.651	0.017	3.59	1.61–8.02	**0.006**
Depression	18 (24.3)	10.201	0.023	2.69	1.53–4.72	**0.001**
ADD/ADHD	2 (2.7)	0.184	0.000	0.74	0.18–3.12	0.668
Other ^a^	5 (6.8)	1.661	0.004	1.98	0.76–5.18	0.198
**Mental Health Medications**						
General Categories						
*Any Mood or Neurological*	20 (27.0)	7.512	0.017	2.22	1.30–3.81	**0.006**
*Mood Only*	20 (27.0)	10.638	0.024	2.63	1.53–4.52	**0.001**
*ADD/ADHD only*	2 (2.7)	0.124	0.000	0.78	0.18–3.29	0.725
*Both Mood and ADD/ADHD*	2 (2.7)	3.448	0.008	1.42	1.01–1.99	0.063
Specific Types						
*Anti-anxiety/panic*	5 (6.8)	3.960	0.009	3.09	1.15–8.28	**0.047**
*Anti-depressant*	18 (24.3)	10.544	0.024	2.739	1.56–4.81	**0.001**
SSRI	17 (23.0)	13.502	0.030	3.297	1.84–5.90	**<0.001**
NDRI	1 (1.4)	0.107	0.000	0.725	0.10–5.47	0.744
SNRI	1 (1.4)	0.149	0.000	1.538	0.19–12.18	0.700
Other ^b^	1 (1.4)	1.473	0.003	5.160	0.53–50.22	0.225
*Anticonvulsant*	1 (1.4)	0.149	0.000	1.538	0.19–12.18	0.700
*Antipsychotic*	0 (0)	0.634	0.001	0.000	0.00–	0.426
*Other ^c^*	0 (0)	0.888	0.002	0.000	0.00–	0.346

Abbreviations: ADD/ADHD = attention deficit disorder/attention deficit hyperactivity disorder; CI = confidence interval; NDRI = norepinephrine dopamine reuptake inhibitor; OR = odds ratio; SNRI = serotonin norepinephrine reuptake inhibitor; SSRI = selective serotonin reuptake inhibitor. Notes: Bold denotes significant *p* value. ^a^ Includes chronic migraine and/or headache, unspecified seizure, epilepsy, obsessive-compulsive personality disorder, schizophrenia/bipolar, cerebral palsy, Chiari malformation, and syringomyelia. ^b^ Includes tricyclic anti-depressants, trazodone, and unspecified anti-depressants. ^c^ Includes triptans, serotonin agonists, and unspecified others.

**Table 4:** Frequency of other non-modifiable risk factors and associations to previously experiencing EAH.

	N (%) (x/74 EAH)	χ^2^	R^2^	OR	95% CI	*p* Value
**Any Medical Condition**	38 (51.4)	4.072	0.009	1.63	1.02–2.61	**0.044**
Previous EHI	58 (79.4)	25.775	0.057	3.80	2.16–6.69	**<0.001**
Kidney	2 (2.7)	5.920	0.013	15.71	2.18–113.14	**0.015**
Cardiovascular	10 (13.5)	4.821	0.011	2.37	1.17–4.82	**0.028**
*Bleeding disorder*	2 (2.7)	1.643	0.004	3.12	0.67–14.50	0.200
*Sickle cell trait/disease*	3 (4.1)	2.735	0.006	3.38	0.95–12.02	0.098
*Heart murmur*	5 (6.8)	0.308	0.001	1.32	0.51–3.39	0.579
*High blood pressure/cholesterol*	7 (9.5)	0.118	0.000	1.16	0.52–2.59	0.731
Gastrointestinal	7 (9.5)	1.659	0.004	1.77	0.78–4.03	0.198
Respiratory	22 (29.7)	1.647	0.004	1.41	0.84–2.37	0.199
*Asthma*	5 (6.8)	0.067	0.000	1.14	0.44–2.91	0.795
*Smoking*	4 (5.4)	0.292	0.001	1.35	0.47–3.86	0.589
Metabolic	1 (1.4)	0.326	0.001	0.58	0.08–4.36	0.568
Diabetes	0 (0)	2.039	0.005	0.00	0.00–	0.153
Chronic inflammatory disease	0 (0)	3.586	0.008	0.00	0.00–	0.058
Other chronic illness ^a^	5 (6.8)	0.002	0.000	1.02	0.40–2.61	0.963
Diagnosed ED	5 (6.8)	3.129	0.007	2.66	1.00–7.08	0.077
**Any Prescription Medication**	37 (50.0)	1.917	0.004	1.40	0.97–2.23	0.166
Cardiovascular	7 (9.5)	1.67	0.004	2.48	0.72–8.57	0.196
Asthma	5 (6.8)	0.067	0.000	1.14	0.44–2.91	0.795
Allergy	6 (8.1)	0.406	0.001	1.34	0.56–3.20	0.524
Diabetes	0 (0)	2.039	0.005	0.00	0.00–	0.153
Gastrointestinal	1 (1.4)	0.000	0.000	1.02	0.13–7.84	0.984
Hormonal	17 (23.0)	0.028	0.000	0.95	0.55–1.67	0.866
Immunomodulating	0 (0)	1.016	0.002	0.00	0.00–	0.314
Metabolic	1 (1.4)	0.326	0.001	0.58	0.08–4.36	0.568
NSAIDs	2 (2.7)	2.549	0.006	4.47	0.91–21.90	0.110
Thyroid	1 (1.4)	0.093	0.000	1.40	0.18–10.97	0.761
**Nutritional Behaviors**						
Pressure to change weight	33 (44.6)	1.725	0.004	1.38	0.86–2.21	0.189
Target weight set/routine weights	7 (9.5)	0.037	0.000	0.92	0.42–2.06	0.847

Abbreviations: EAH = exercise-associated hyponatremia; EHI = exertional heat illness; NSAID = non-steroidal anti-inflammatory drug; OR = odds ratio. Notes: Bold denotes significant *p* value. ^a^ Includes autoimmune disorders, lupus, arthritis, anemia, psoriasis, Raynaud’s, hemophilia, and unspecified other chronic illnesses.

**Table 5:** Frequency and association to sex ^a^ for significant EAH risk factors among MB artists.

	Sex N/X Reporting Risk Factor (%)			Regression				
	Male	Female	Other ^b^	χ^2^	R^2^	OR	95% CI	*p* Value
**Medical conditions**								
Any mental health (n = 207)	52 (25.1)	153 (73.6)	3 (1.5)	28.783	0.039	2.197	1.64–2.95	**<0.001**
Anxiety (n = 45)	7 (15.6)	37 (82.2)	1 (2.2)	14.495	0.044	2.902	1.69–5.00	**<0.001**
Depression (n = 139)	28 (20.1)	108 (77.7)	3 (2.1)	31.573	0.051	2.653	1.86–3.78	**<0.001**
Previous EHI (n = 611)	196 (32.1)	410 (67.1)	5 (0.8)	55.349	0.060	2.405	1.90–3.04	**<0.001**
Any medical (n = 484)	189 (39.0)	289 (59.7)	6 (1.2)	3.210	0.004	1.221	0.98–1.52	0.073
Cardiovascular (n = 80)	30 (37.5)	49 (61.3)	1 (1.3)	0.671	0.001	1.196	0.78–1.83	0.413
Kidney (n = 4)	1 (25.0)	3 (75)	0 (0)	0.359	0.007	1.744	0.29–10.43	0.549
**Medications and supplements**								
Any mood or neurological (n = 182)	42 (23.1)	137 (75.3)	3 (1.6)	31.016	0.044	2.370	1.732–3.25	**<0.001**
Mood only (n = 160)	32 (20.0)	125 (78.1)	3 (1.9)	36.504	0.055	2.703	1.93–3.79	**<0.001**
Anti-anxiety (n = 31)	5 (16.1)	25 (80.6)	1 (3.2)	10.745	0.042	2.953	1.57–5.54	**0.001**
Anti-depressant	27 (19.7)	107 (78.1)	3 (2.2)	32.334	0.052	2.703	1.89–3.86	**<0.001**
SSRIs (n = 111)	22 (19.8)	86 (77.5)	3 (2.7)	26.642	0.048	2.668	1.82–3.92	**<0.001**
Other supplements (n = 59)	24 (40.7)	35 (59.3)	0 (0)	0.001	0.000	1.009	0.62–1.66	0.970
**Nutritional behaviors**								
Perceived ED (n = 120)	30 (25.0)	89 (74.2)	1 (0.8)	13.335	0.023	1.941	1.36–2.78	**<0.001**
Diet pills to change weight (n = 30)	5 (16.7)	25 (83.3)	0 (0)	6.326	0.025	2.349	1.23–4.50	**0.012**
Dieting (n = 276)	96 (34.8)	180 (65.2)	0 (0)	4.845	0.006	1.331	1.03–1.72	**0.028**

Abbreviations: CI = confidence interval; EAH = exercise-associated hyponatremia; ED = eating disorder; MB = marching band; SSRI = selective serotonin reuptake inhibitors. Note: Bold denotes significant *p* values. ^a^ Regression analysis conducted for sex and risk factor with male as reference group. ^b^ Includes male-to-female, female-to-male, and prefer not to disclose; no individuals identifying within this group reported previously experiencing exercise-associated hyponatremia.

## Data Availability

The raw data supporting the conclusions of this article will be made available by the authors upon request.
